# Systematic Engineering for Improved Carbon Economy in the Biosynthesis of Polyhydroxyalkanoates and Isoprenoids

**DOI:** 10.3390/ma11081271

**Published:** 2018-07-24

**Authors:** Huibin Zou, Tongtong Zhang, Lei Li, Jingling Huang, Nan Zhang, Mengxun Shi, He Hao, Mo Xian

**Affiliations:** 1 College of Chemical Engineering, Qingdao University of Science and Technology, Qingdao 266042, China; zttyqz@outlook.com (T.Z.); lilei0924@hotmail.com (L.L.); huangjingling12@163.com (J.H.); zhangnan195@outlook.com (N.Z.); shimengxun@163.com (M.S.); hehaoqust@163.com (H.H.); 2 CAS Key Laboratory of Bio-based Materials, Qingdao Institute of Bioenergy and Bioprocess Technology, Chinese Academy of Sciences, Qingdao 266101, China; xianmo@qibebt.ac.cn

**Keywords:** biosynthesis, carbon economy, polyhydroxyalkanoates, isoprenoids

## Abstract

With the rapid development of synthetic biology and metabolic engineering, a broad range of biochemicals can be biosynthesized, which include polyhydroxyalkanoates and isoprenoids. However, some of the bio-approaches in chemical synthesis have just started to be applied outside of laboratory settings, and many require considerable efforts to achieve economies of scale. One of the often-seen barriers is the low yield and productivity, which leads to higher unit cost and unit capital investment for the bioconversion process. In general, higher carbon economy (less carbon wastes during conversion process from biomass to objective bio-based chemicals) will result in higher bioconversion yield, which results in less waste being generated during the process. To achieve this goal, diversified strategies have been applied; matured strategies include pathway engineering to block competitive pathways, enzyme engineering to enhance the activities of enzymes, and process optimization to improve biomass/carbon yield. In this review, we analyze the impact of carbon sources from different types of biomass on the yield of bio-based chemicals (especially for polyhydroxyalkanoates and isoprenoids). Moreover, we summarize the traditional strategies for improving carbon economy during the bioconversion process and introduce the updated techniques in building up non-natural carbon pathways, which demonstrate higher carbon economies than their natural counterparts.

## 1. Introduction

When compared with traditional synthetic chemistry, biosynthesis has several defining features [1,2]: (1) “Protective” and “deprotected” steps during chemical synthesis are not included, which can reduce the potential for impurities in final products; (2) higher selectivity in the synthesis of chemicals with complex structures, such as artemisinin [3] and paclitaxel [4]; and, (3) biosynthesis usually uses renewable substrates, which can reduce the dependence on non-renewable resources. However, current chemical industries still rely on chemical routes and fossil-based resources with well understood technologies and significant advantages in economies of scale. Comparatively, most of the biotechnologies need considerable efforts in achieving economic viability [5]. One of the often-seen barriers of biosynthesis is the low yield and productivity, which leads to higher unit cost and unit capital investment.

With a goal of “efficient synthesis”, chemists and biologists are developing novel methods, techniques and strategies for improved “atomic economy” in synthesis, which aim to maximize the use of atoms from reactants or substrate. The conception of “atomic economy” was promoted as early as 1991, and “carbon economy” aims to (1) maximize the use of carbon atoms from the raw material molecules into the final products; and, (2) to reduce the carbon waste and pollution during production process [6]. In the case of biosynthesis, rational engineering of the carbon flux pathway is the typical strategy to improve biosynthesis [7,8].

Systematic engineering efforts have been made to improve the biosynthesis efficiency of value added bioproducts, like isoprenoids and polyhydroxyalkanoates (PHAs). Isoprenoids include highly diversified nature products and secondary metabolites, and they can be biosynthesized through two pathways, the mevalonate pathway and the MEP (methylerythritol phosphate) pathway [9] from the precursors of isopentenyl phosphate (IPP) and dimethylallyl pyrophosphate (DMAPP) [10]. PHA is a general term for linear polyesters composed of 3-hydroxy fatty acids and a broad range of PHAs can be microbially produced as high-performant biomaterials [11].

**Figure 1:**
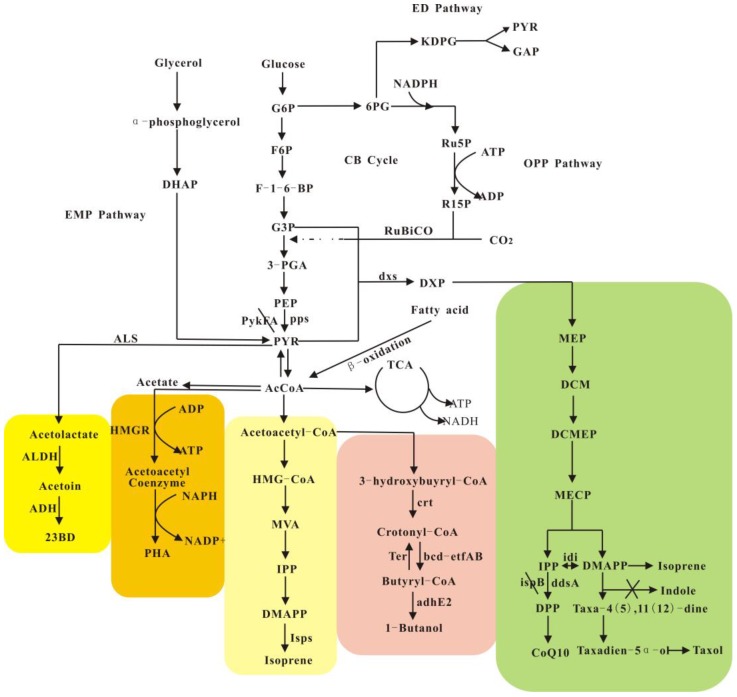
Highly complex metabolic pathways for the biosynthesis of representative bio-based products (with difference colors) from variable substrates like glucose, glycerol, fatty acids and CO_2_. PEP: phosphoenolpyruvate; PYR: pyruvate; KDPG: 2-keto-3-deoxy-6-phosphogluconate; 6PG: 6-phosphogluconate; 3-PGA: 3-phosphoglycerate; R15P: ribulose-1,5-bisphosphate; R5P: ribulose-5-phosphate; ALDC: acetolactate decarboxylase; ADH, alcohol dehydrogenase; DXP: 1-deoxy-d-xylulose 5-phosphate; IPP: isopentenyl diphosphate; DMAPP: dimethylallyl diphosphate; DPP: decaprenyl diphosphate; ddsA: encoding decaprenyl diphosphate synthase; pps: encoding PEP synthase; DHAP: dihydroxyacetone phosphate; GAP: glyceraldehyde-3-phosphate; G6P: glucose-6-phosphate; F-1,6-BP: fructose-1,6-bisphosphate; AcCoA: acetyl-CoA; NADH: nicotinamide adenine dinucleotide; MVA: mevalonate; TCA: tricarboxylic acid cycle; ATP: adenosine triphosphate; (1) RuBisCO: ribulose-1,5-bisphosphate carboxylase/oxygenase; (2) ALS: acetolactate synthase; (3) *dxs*: encoding DXP synthase; (4) *pykFA*: encoding pyruvate kinase isoenzymes I and II; (5) *pps*: encoding PEP synthase; (6) *ispB*: encoding octaprenyl diphosphate synthase; (7) *ddsA*: encoding decaprenyl diphosphate synthase; (8) *idi*: encoding IPP isomerase; (9) crt (CA) (10) Ter: trans-enoyl-CoA reductase; (11) bcd-etfAB: butyryl-CoA dehydrogenase complex; (12) adhE2 (13) HMGR: malonyl-CoA reductase from *Chloroflexus aurantiacus*; and, (14) *Isps*: Isoprene synthases.

In case of isoprenoid biosynthesis by engineered *Escherichia coli* (Figure 1), the knockout of *pykFA* along with the over expression of *pck* maintains carbon flux homeostasis between glyceraldehyde 3-phosphate and pyruvate, and ultimately increases the production of coenzyme Q10 [12]. The addition of the genes *ispB* with *ddsA* from *Gluconobacter suboxydans* can further increase carbon flux towards coenzyme Q10 [12]. Similarly, this strategy (deleting *pykFA* and overexpressing *pck*) was also effective to increase the bioproduction of lycopene [13]. Systematic engineering efforts have also been made in yeast cell factories [14,15,16]. For example, the GAL regulatory system is the most common expression system in *Saccharomyces cerevisiae*. The *GAL* promoter is transcribed by the activation of the Gal4p protein, whereas Gal80p, as an inhibitor, binds to Gal4p and reduces the transcription level of the *GAL* promoter. Torchia et al. modified the GAL-inducible expression system by knocking out the *Gal80* gene, which blocked galactose-induced signaling, and allowed the increased transcription of the *GAL* promoter in yeast strains [17,18,19]. The modified GAL system can be applied in the biosynthesis of isoprenoids by yeast strains, as the bottleneck-enzymes in isoprenoid pathway usually demand over expression. For example, the modified GAL system can effectively increase the transcriptional level of Isps (key enzyme in isoprene production); and, the production of isoprene was increased from 6.0 to 23.6 mg/L through shake flask fermentation [20].

Central carbon metabolism can also be affected by physiological conditions during the feeding process [21]. For example, the limitation of S/N/C in the growth medium could affect the composition of proteins [21,22], whereas adjusting the C/N ratio during different growing stages of *Ralstonia eutropha* could significantly increase carbon flux towards polyhydroxyalkanoate (PHA) production [23]. Other physiological conditions, such as temperature, substrate type, and feeding strategy, also affect carbon metabolism [21].

**Figure 2:**
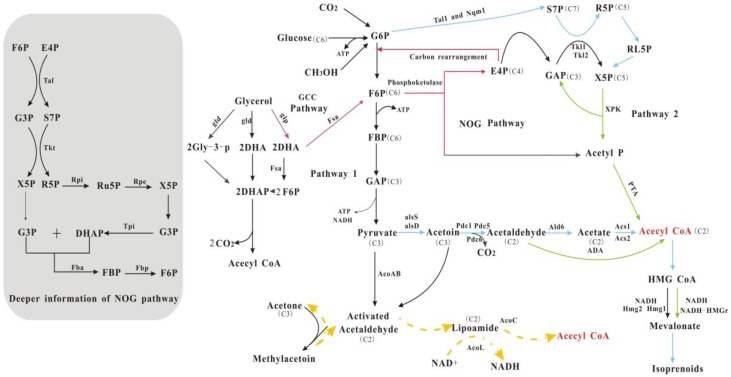
Rewired carbon pathways with improved carbon economy. G6P: 6-P-glucose; F6P: fructose-6-phosphate; FBP: fructose 1,6-bisphosphatase; E4P: erythorse 4-phosphate; G3P: glyceraldehyde 3-phosphate; X5P: xylulose 5-phosphate; R5P: ribose 5-phosphate; Ru5P: ribulose 5-phosphate; S7P: sedoheptulose 1,7-bisphosphatase; RL5P: Ribulose-5-phosphate; DHA: dihydroxyacetone; Gly-3P: glycerol 3-phosphate; alsS: acetolactate synthase; alsD: acetolactate decarboxylase; acoAB: acetoin:2,6-dichlorophenolindophenol oxidoreductase; acoC: dihydrolipoamide acetyltransferase; acoL: dihydrolipoamide dehydrogenase; Tal: transaldolase ; Tkt: transketolase; Rpi: ribose-5-phosphate isomerase; Rpe: ribulose-5-phosphate epimerase; Tpi: triose phosphate isomerase; Fba: FBP aldolase; Fbp: fructose 1,6-bisphosphatase; glp: glycerol kinase; gld: glycerol dehydrogenase; and, fsa: fructose-six-phosphate aldolase.

A recently applied strategy to improve the carbon economy of biosynthetic pathways is the rewiring of natural carbon pathways. Through the rewiring strategies, unnatural biosynthetic pathways are developed with improved theoretical carbon yield (Figure 2). For example, an unnatural NOG (non-oxidative glycolysis) pathway was constructed to redirect carbon flux from sugars to acetyl-CoA [24]; the NOG pathway showed higher carbon economy when compared with the natural oxidative glycolysis pathway. Here, we present a review of these traditional and updated strategies that have been proposed from studies at many different levels. Moreover, we propose some general rules for the improvement of carbon economy in biosynthesis.

## 2. Starting Point: Carbon Economies Using Different Substrates in Biosynthesis

### 2.1. The Biosynthesis of PHA from Different Carbon Sources

Polyhydroxyalkanoates (PHA) constitute one of the commercialized bio-products and are often utilized as high-performant biomaterials [11]. The scale and benefits of industrial PHA production have largely relied on the cost of carbon sources [25,26,27], as the cost of carbon sources accounted for 28–50% of total PHA production costs [28,29]. Therefore, a broader range of substrates have been explored for PHA production. In this study, the authors analyzed the carbon flux from different substrates (Figure 1) and compared their resultant PHA production levels (Table 1). The majority of PHA products are synthesized while using acetyl-CoA as a building block (Figure 1). Because the natural oxidative pathway for sugars to two acetyl-CoA molecules results in the loss of two carbon atoms, the theoretical yield of acetyl-CoA from oil substrates (through the beta oxidation cycle) should be higher than that obtained from sugar substrates.

Due to the stable supply and quality, glucose and starch are often utilized as the carbon source for PHA fermentation. For example, high-titer polyhydroxybutyrate (PHB) fermentation was achieved by engineered *R. eutropha* while maintaining the glucose feed at 10–20 g/L, which resulted in a final PHB production of 121 g/L. Moreover, by concomitantly adding glucose and propionic acid as dual carbon substrates, the author could generate the co-polymer PHBV (polyhydroxybutyrate-co-hydroxyvalerate) at a concentration of 110 g/L [33]. High-titer PHA production was also achieved by other strains, including engineered *E. coli* [34], *A. chroococcum* [44], and *Bacillus megaterium* [39], while using glucose as the carbon source. Starch sources, including extruded rice bran (ERB) and extruded cornstarch (ECS), have also been used in PHA production. Interestingly, when an ERB:ECS ratio of 1:8 (*w*/*w*) was used as the major carbon source, PHA concentrations of 77.8 g/L could be achieved, whereas when ECS alone was utilized as the main carbon source, PHA titers only reached 24.2 g/L [45]. Waste potato starch has also been utilized as a carbon substrate by engineered *R. eutropha* NCIMB, with the PHB production of 94 g/L [46].

Fatty acids and vegetable oils have also been utilized as carbon sources in the biosynthesis of PHA*. R. eutropha*, which is one of the industrial strains that is used for the biosynthesis of PHA, can efficiently utilize soybean oil as the sole carbon source to produce up to 0.72–0.76 g of PHA per gram of soybean oil [30]; this yield is significantly higher than those that were reported by other studies using non-oil substrates (Table 1). Other oil substrates can also be fed as carbon sources in PHA production (Table 1). For example, discarded vegetable oil can be utilized in PHA production by *Pseudomonas aeruginosa*, resulting in the intracellular PHA production of 37.34% (*w*/*w*) [27].

Non-food carbon substrates from industrial sources can also be utilized as economic carbon sources in PHA production, e.g., glycerol, alcohol, and molasses (Table 1). Glycerol is more often utilized in the biosynthesis of PHA, and engineered *E. coli* strains have shown excellent performance in the production of PHA from glycerol [38]. In a comparative study, *E. coli* showed 25–28 fold higher conversion efficiency of glycerol than the native PHB producer *Streptomyces aureofaciens* [47]. In addition to *E. coli*, other strains, including *Paracoccus denitrificans* and *R. eutropha* [43], have been shown to efficiently produce PHA from glycerol [26]. Rare carbon sources have also been applied in PHA production. For example, poly(3-hydroxyvalerate) can be produced from n-pentanol by the methylotrophic bacterium *P. denitrificans* [48].

### 2.2. Isoprenoid Biosynthesis from Diverse Carbon Sources

Natural isoprene is mainly found in plants [49], which is generated from the C5 subunits DMAPP (dimethylallyl pyrophosphate) and its isomer IPP (isopentenyl pyrophosphate). Highly diversified isoprenoids (terpenes) can be further biosynthesized with increased numbers of C5 subunits [50]. Although variable isoprenoids have been synthesized by engineering their biosynthetic routes using sugar and glycerol substrates (Table 2), only few studies have reported satisfactory concentrations [51,52,53,54,55,56,57], thereby suggesting the low efficiency of isoprenoid production from glucose or glycerol substrates.

In natural biosynthetic pathways of isoprenoids, C5 subunits can be converted from sugar and glycerol substrates via either the MVA (mevalonate) pathway or MEP (2-C-methyl-d-erythritol 4-phosphate) pathway (Figure 1). Both of the pathways have low theoretical yields from glucose and glycerol substrates. Through the MEP pathway, each mole of C5 building block requires 1.25 mol of glucose or 2.5 mol of glycerol; through the MVA pathway, each mole of C5 building block requires 1.5 mol of glucose or 3 mol of glycerol [50]. Carbon economy will be further decreased for isoprenoids with large numbers of C5 precursors in the structure.

If fatty acids and oils are used as the carbon source, the theoretical yield would be higher than those of sugar and glycerol, as fatty acids can be converted into C2 building blocks (acetyl-CoA) by beta-oxidation without significant carbon loss, after which the acetyl-CoA precursor can be converted to C5 building blocks through the MVA pathway (Figure 1). In this integrated beta-oxidation/MVA pathway, one mole of stearic acid (C18) can produce three moles of C5 building block [50]. However, few studies have reported the isoprenoid biosynthesis from oil or fatty acid substrates. Our lab conducted several pioneering studies [58], and has successfully biosynthesized MVA from oil substrates while using engineered *R. eutropha* [59]. Further studies are needed to develop engineered pathways with higher carbon economy for the biosynthesis of isoprenoids.

## 3. Rewiring Carbon Flux Pathways for Improved Carbon Economy

### 3.1. Non-Oxidative Glycolysis Pathway

Natural glycolysis includes the partial oxidation of glucose to pyruvate, which gets subsequently decarboxylated to acetyl-CoA (Figure 2). Through the oxidative glycolysis pathway, two acetyl-CoA molecules can be produced from one glucose molecule. Pioneering studies have aimed to rewire this central carbon metabolic pathway to achieve optimal carbon flux towards objective bio-molecules with less carbon loss [7,8,77]. Using this strategy, a non-oxidative glycolysis pathway (NOG) was constructed [24], which can convert hexose and pentose substrates to C2 metabolites with improved carbon yield. Phosphoketolases play key roles in the NOG pathway. For pentose substrates, phosphoketolases catalyze the cleavage of xylulose 5-phosphate (X5P) and sedoheptulose 7-phosphate (S7P) to acetyl-P, glyceraldehyde 3-phosphate (G3P), and ribose-5-phosphate (R5P); for hexose substrates, F6P (from hexose substrates) is converted to acetyl-P and erythrose-4-phosphate (E4P). G3P, R5P, and E4P can be further recycled through the NOG pathway in order to produce additional acetyl-P molecules (Figure 2), and acetyl-P can be converted to acetyl-CoA for downstream biosynthesis pathways. Thus, complete carbon conservation in hexose/pentose catabolism to acetyl-CoA can be achieved while using the artificial NOG pathway [24], and the engineered NOG pathway could allow for the improved biosynthesis of acetyl CoA derived bio-products. For example, engineering the NOG pathway can significantly improve the yield of P(3HB) [78].

### 3.2. Engineering Yeast Central Pathways to Improve Carbon Economy

As summarized in Table 2, engineered *S. cerevisiae* is often utilized for the biosynthesis of isoprenoids [79,80]. However, the limitations of the natural central pathway lead to the low yield and productivity in most cases (Table 2). Rewiring yeast central carbon metabolism is a feasible strategy, which could improve the theoretical yield of bio-products. A recent study thoroughly engineered the central metabolism of *S. cerevisiae* for the production of farnesene from glucose [81]. Using insight from the NOG pathway that is described above, heterologous phosphoketolases were also engineered in this study to reduce the loss of carbon. Moreover, this study further engineered ADA (aldehyde dehydrogenase CoA-acylating) and NADH-HMGr (NADH-specific HMG-CoA reductase) to achieve a balanced metabolic network (e.g., reduced ATP consumption and improved redox balance). These systematic engineering efforts (Figure 2) significantly improved the theoretical yield of farnesene from glucose. More importantly, the results showed that, when compared with control strains, strains expressing this rewired central pathway produced 25% more farnesene while requiring 75% less oxygen. It can be speculated that the strategy can be applied in the biosynthesis of a broader range of acetyl CoA derived bio-products with improved carbon economy [24].

### 3.3. Acetone as a Carbon Source: Engineering the Methylacetoin Pathway

One of the strategies to improve the carbon economy in biosynthesis is to utilize inexpensive organic chemicals of industrial origin as sole or co-substrates. In this context, the development of artificial metabolic pathways is an essential prerequisite for the successful conversion of unnatural carbon substrates to the desired bio-products or intermediates that could be further converted to the desired products. Acetone is such a candidate. Inspired by the degradation pathway of methylacetoin [82,83], a pioneering study has successfully constructed a carbon flux pathway for the biosynthesis of methylacetoin while using the substrates acetone and glucose [84], and each mole of methylacetoin can be synthesized from 0.5 mol of glucose and one mole of acetone. Methylacetoin and its derivatives can be utilized as fuels [85] and chemical building blocks for the synthesis of solvents, polymers [86], and pharmaceutical products [87,88]. Furthermore, methylacetoin can be converted into methyl isopropenyl ketone, which can be applied in the production of optical plastics, photodegradable plastics, and fire-resistant rubbers [84]. As methylacetoin can be further metabolically converted to acetyl-CoA and pyruvate, acetone can also be linked to central carbon metabolism via the artificial pathway (Figure 2) in the production of broader bioproducts.

## 4. Process Optimization for Maximum Carbon Flux towards Bioproducts

The rational design and rewiring of natural carbon pathways will improve the theoretical carbon economy. In addition, systematic process optimization should be conducted to achieve maximum yields from substrates to the desired bio-products via these designed pathways, while maintaining the balance of energy and redox.

Process optimization often aims to improve the catalytic efficiency of key enzymes in the metabolic pathway. High-performance enzymes are often screened during the optimization process, and their expression levels are also optimized. For example, isoprene synthase (IspS) is a bottleneck enzyme that catalyzes the synthesis of isoprene from the isoprenoid building block DMAPP. Earlier studies identified the isoprene synthase gene in poplar and kudzu [49,89], but their natural catalytic activities are relatively low [90]. Other studies isolated *IspS* from sweet potatoes, and this variant showed higher efficiency than that of the poplar *IspS* [91].

Moreover, the systematic balancing of the expression levels of key enzymes is often a focus of process optimization. For example, the traditional process for the production of the anti-cancer drug paclitaxel [92] is through the extraction from the pacific yew tree; however, the yield is quite low [93,94]. The development of a semi-synthetic process with higher yield was proposed by the extraction of baccatin III from cultured plant cells, followed by the chemical synthesis of paclitaxel from baccatin III [95,96]. Moreover, a multi-modular biosynthetic process was developed that divided the natural paclitaxel pathway into several sub-level pathways, and the optimized process resulted in improved yield in the production of paclitaxel and its analogs [54]. The characteristics of key enzymes in the paclitaxel pathway facilitated further optimization [69,97,98]. Specifically, the expression level of the key enzymes dxs, idi, ispD, and ispF (detailed information is shown in Figure 1) in the upper pathway and GGPP synthase and taxadiene synthase in the downstream pathway was systematically balanced to avoid the generation of the intermediate indole, which significantly improved the yield of paclitaxel [99]. This strategy was also utilized to optimize the biosynthetic process for the production of taxane. After process optimization, the yield of the intermediate taxane diene was increased 15,000-fold [99], and the yield of the paclitaxel-5α-alcohol was increased 2400-fold [100].

Fermentation process optimization is another strategy to improve the productivity of objective bio-products, as is often seen in the fermentation process of coenzyme Q10 [101,102], which is also derived from the isoprenoid pathway (Figure 1). It was reported that the yield of coenzyme Q10 is affected by variations in growth media [103], and SOB (Super optimal broth) medium showed the highest yield. Further study confirmed that the SOB medium promotes the expression of genes (*dxs*, *idi*, *pck*, *ispA*) that code key enzymes in the biosynthesis of CoQ10 [104]. Moreover, CoQ10 production can be increased when the initial pH is maintained at 5.5 [103].

## 5. Conclusions

From a biotechnological point of view, improved carbon economy in biosynthesis correlates with decreased unit cost of bio-products, which increases their compatibility with potential markets. The traditional saccharide media and substrates that are used in biosynthesis have been optimized for many years to achieve maximum microbial growth, product yields, and minimum costs, so substrate and media optimization is often seen in the industrial fermentation processes (as summarized in Table 1 for PHA fermentation). However, traditional strategies in the optimization of fermentation processes may not meet the needs for value-added and structurally complex bio-products (as indicated in Table 2 for isoprenoid biosynthesis).

Synthetic biologists are also focused on overcoming the low theoretical carbon yields and have focused on expanding the diversity of candidate pathways with improved carbon economies. Synthetic biologists have also developed artificial pathways, as shown in pioneering studies that have rationally rewired carbon metabolic pathways that are not found in nature [24,81]. This area of research is rapidly developing: a seemingly infinite number of novel substrates, enzymes, pathway information, and novel bio-products are under investigation. Clearly, more frontier techniques and interdisciplinary discoveries of major importance wait around the corner.

## Figures and Tables

**Table 1:** Microbial production of polyhydroxyalkanoates (PHA) from variable carbon substrates.

Carbon Substrate	Strain	Production ^a^	Yield ^b^	Reference
Soybean oil	*R* *. eutroph* *a*	95.8 g/L	0.76 g/g	[30]
Soybean oil	*R* *. eutroph* *a*	102.1 g/L	0.72 g/g	[30]
Fructose	*R* *. eutroph* *a*	6.5 g/L	0.32 g/g	[31]
Xylose	*R* *. eutroph* *a*	0.2 g/L	0.04 g/g	[31]
Lactic acid	*R* *. eutroph* *a*	2.2 g/L	0.22 g/g	[31]
Propionic acid	*R* *. eutroph* *a*	1.6 g/L	0.16 g/g	[31]
PG	*R* *. eutroph* *a*	31.7 g/L	0.36 g/g	[26]
GRP	*R* *. eutroph* *a*	19.1 g/L	0.34 g/g	[26]
Glucose/P	*R. eutropha*	40.7 g/L	0.35 g/g	[32]
Glucose/CA	*R. eutropha*	17.2 g/L	0.17 g/g	[32]
Glucose	*R. eutropha*	92.0 g/L	N/A	[33]
Glycerol	*R* *. eutroph* *a*	25.8 g/L	0.84 g/g	[26]
Glucose	*R* *. eutroph* *a*	121.0 g/L	N/A	[33]
Glucose	*E. coli*	1.9 g/L	0.17 g/g	[34]
Glucose	*E. coli*	128.6 g/L	0.23 g/g	[35]
Xylose	*E. coli*	1.9 g/L	0.19 g/g	[36]
L-arabinose	*E. coli*	1.0 g/L	0.15 g/g	[36]
Lactose	*E. coli*	1.0 g/L	0.12 g/g	[36]
Molasses	*E. coli*	31.6 g/L	0.29 g/g	[37]
Glycerol	*E. coli*	11.0 g/L	0.5 g/g	[38]
Molasses	*B. megaterium*	30.6 g/L	0.08 g/g	[39]
Starch	*A. chroococcum*	25.0 g/L	0.25 g/g	[40]
Sucrose	*R. eutropha*	98.7 g/L	0.11 g/g	[41]
Tapioca	*R. eutropha*	61.5 g/L	0.25 g/g	[42]
n-Pentanol	*P* *.* *denitrificans*	1.2 g/L	N/A	[43]

^a^: Grams of products per liter fermentation broth (g/L); ^b^: grams of products per gram of carbon substrate; N/A: the information is not available.

**Table 2:** Microbial production of representative isoprenoids from variable carbon substrates.

Isoprenoids	Carbon Substrate	Strain	Production ^a^	Reference
Taxadiene	Glucose + Glycerol	*E. coli*	1.0 g/L	[55]
	Glucose	*S. cerevisiae*	8.7 mg/L	[60]
α-santalene	Glucose	*S. cerevisiae*	0.2 mg/L	[61]
Amorphadiene	methylerythritol	*E. coli*	24 mg/L	[62]
	Glucose	*S. cerevisiae*	153 mg/L	[63]
	Glycerol	*E. coli*	293 mg/L	[64]
	Galactose + Glucose	*S. cerevisiae*	40 g/L	[65]
Astaxanthin	Pyruvate	*E. coli*	1.4 mg/g	[66]
Levopimaradiene	glyceraldehyde3-phosphate + pyruvate	*E. coli*	700 mg/L	[67]
Militradiene	Glucose	*S. cerevisiae*	365 mg/L	[68]
β-carotene	Pyruvate + Glyceraldehyde 3-phosphate	*E. coli*	6 mg/g	[69]
Patchoulol	Galactose	*S. cerevisiae*	40.9 mg/L	[70]
Carotenoids	Glucose	*E. coli*	1.4 mg/L	[53]
Lycopene	Glucose	*E. coli*	7.8 mg/g	[71]
	Glucose	*E. coli*	12.3 mg/g	[72]
	Glucose	*E. coli*	1.0 mg/g	[51]
Artemisinin	Glucose	*S. cerevisiae*	100 mg/L	[63]
Amorphadiene	Glucose	*S. cerevisiae*	20 mg/L	[73]
Diterpene	Glucose or Glycerol	*E. coli*	700 mg/L	[67]
Zeaxanthin	Tagetes erecta’s red flowers 23% dry weight	*E. coli*	1.6 mg/g	[74]
	Glucose + Casamino Acids	*E. coli*	820 µg/g	[75]
Lycopene	Glucose	*E. coli*	12.3 mg/L	[72]
	Glucose	*E. coli*	16 mg/g	[76]

^a^: Mass of products per liter fermentation broth (g/L) or per cellular dry weight (g/g).
